# Partner’s problematic social media use, woman’s time perspective, and prenatal depression

**DOI:** 10.1007/s00737-024-01482-w

**Published:** 2024-06-15

**Authors:** Małgorzata Sobol, Agata Błachnio, Inna Hryhorchuk, Elzbieta Plucinska, Janusz Stasiniewicz, Aneta Przepiórka

**Affiliations:** 1 Department of Psychology, University of Warsaw, ul. Stawki 5/7, Warsaw, 00-183 Poland; 2 John Paul II Catholic University of Lublin, al. Raclawickie 14, Lublin, 20-950 Poland; 3 Żywiec Hospital, Pola Lisickich 80, Żywiec, 34-300 Poland

**Keywords:** Problematic social media use, Pregnancy, Time perspective, Depressive symptoms, Fatalism

## Abstract

**Purpose:**

Using social media can have negative consequences. The present study aimed to examine how the partner’s problematic social media use (SMU) was related to the pregnant woman’s time perspective and prenatal depression.

**Methods:**

The study included 30 pregnant women and their 30 male partners. Research was conducted twice: in the first and third trimesters of pregnancy. Women completed online measures: the Zimbardo Time Perspective Inventory Fatalism scale (ZTPI-Fat), the Dark Future Scale (DFS), and the Edinburgh Postpartum Depression Scale (EPDS). Men completed the online Social Media Addiction Questionnaire (SMAQ).

**Results:**

The woman’s depressive symptoms were positively associated with fatalism (*r* = .35, *p* < .01 in the first trimester; *r* = .49, *p* < .01 in the third trimester) and future negative perspective (*r* = .33, *p* < .05 in the first trimester; *r* = .77, *p* < .001 in the third trimester). Moreover, in the third trimester, women’s depressive symptoms correlated positively with their partners’ problematic SMU (*r* = .36, *p* < .05) and negatively with their financial situation (*r* = − .37, *p* < .05). The results of the mediation analyses showed that the more intensive the partner’s problematic SMU, the stronger the pregnant woman’s fatalism and, consequently, the stronger her future negative perspective, resulting in more severe prenatal depressive symptoms in the third trimester (indirect effect: β = .16, *SE* = .09, 95% CI [.021, .393]).

**Conclusions:**

Our findings show how important the behavior of the partner is for the mental health of the pregnant woman. The results suggest a possible mechanism explaining the relationship between the partner’s problematic SMU and the woman’s prenatal depressive symptoms. This mechanism probably consists in increasing the woman’s sense of helplessness and loss of control over life, which leads to intensified future anxiety and, consequently, to depressive symptoms. Moreover, we interpreted the results to mean that the partner’s time-consuming preoccupation with SMU may make the woman feel emotionally neglected. The lack of support from the partner may give rise to feelings of powerlessness, and may cause depressive symptoms.

Pregnancy is a time of many positive experiences and feelings, but it is also a particularly demanding period, including stressful experiences and negative emotions. The prevalence of maternal prenatal depression in different studies ranged from 8.5 to 31.1% depending on the country (Yin et al. 2021). Prenatal depressive symptoms are the feelings of sadness, blaming oneself, unwarranted anxiety, a sense of failure to cope, anxiety about the future, especially about the child’s uncertain future, which are present during the two weeks and which are a change from hitherto functioning (American Psychiatric Association 2013; Cox 2019). The emotional state of a pregnant woman has a significant impact on the course of pregnancy, the quality of childbirth and the health of the child (Hassan et al. 2023, 2024; Min et al. 2023). Maternal prenatal depression can have a lasting adverse effect on the child’s neurological, behavioral, and emotional development (Chen et al. 2023; Ncube et al. 2017).

With all the problems she is facing, a pregnant woman is particularly strongly in need of support from her partner. Research has revealed negative associations of perceived partner support with the woman’s pregnancy-related anxiety and pregnancy depression (Cheng et al. 2016; Ngai and Ngub 2015). However, the period of pregnancy is an important life challenge not only for the woman but also for her partner. The partner may feel confused and anxious about the new role he will have to take on. The stress and anxiety experienced by the partner may increase escape behavior as his response to the demanding situation. One of the most easily accessible forms of escaping from the difficulties of everyday life is social media use (SMU) (Griffiths et al. 2014; Peng and Liao 2023). Using social media can be useful and enjoyable, but it also has many negative consequences (Casale et al. 2023). The basic characteristics of problematic SMU are difficulties in controlling the use of social media, thinking obsessively about social media, difficulties in controlling the need to use social media and a preference for contacting people online rather than face-to-face (Svicher et al. 2021; Peng and Liao 2023).

A body of research has shown a relationship between problematic SMU and depressive symptoms (Muskens et al. 2023) and low quality of users’ social relationships, including romantic ones (Roberts and David 2023). There are also findings showing that partner’s smartphone use in the pregnant woman’s presence was related to the woman’s depressive symptoms in late pregnancy (Tian and Li 2022). To our knowledge, there has been no research to date on how the partner’s problematic SMU is related to the pregnant woman’s mood. Addressing this issue is very important due to the enormous importance that the partner’s behavior has on the pregnant woman’s mental state and the high frequency of using social media in the modern world.

The present study aimed to examine the association of the partner’s problematic SMU with the pregnant woman’s depressive symptoms. We expected that the relationship between these variables would be explained by the woman’s time perspective. The proposed model of relationships is based on the concept of pregnancy as one of the most difficult changes in life (Cowan and Cowan, 2000) and on the time perspective theory developed by Zimbardo and Boyd (2008). In the period of important life changes, attitudes towards time, especially towards the unknown future, and a sense of control over the changing life situation are of particular importance (Zimbardo et al. 2012). Emotional attitude towards and the degree of focus on the past, present, and future are referred to as time perspective (Zimbardo and Boyd, 2008). Several types of time perspective are distinguished, including future negative time (Zaleski et al. 2019) and present fatalistic perspective (Zimbardo and Boyd, 2008). Future negative perspective is a tendency to focus on the negatively evaluated future (Zaleski et al. 2019). Fatalism consists in passivity and living in anticipation of what fate will bring (Zimbardo & Boyd, 2008). Both future negative perspective and fatalism correlate significantly and positively with depressive symptoms (Diaconu-Gherasim et al. 2023; Zimbardo and Boyd, 2008). We assumed that if the pregnant woman is aware of how much her partner is absorbed by SMU, she will feel helpless and will see herself as having no influence on what may happen. A sense of helplessness, perceived lack of control over one’s life, and the fear of future threats can lead to the onset of depressive symptoms (Zimbardo and Boyd, 2008).

We formulated the following hypothesis: The partner’s higher problematic social media use is associated with the pregnant woman’s higher depressive symptoms, and this association is explained by the pregnant woman’s fatalism and future negative perspective.

## Method

### Participants

The participants were pregnant women (*n* = 30), aged 21–43 years (*M* = 32.28, *SD* = 4.96) and their male partners (*n* = 30) aged 23–52 years (*M* = 34.78, *SD* = 5.19). All participants were Polish. Twenty-seven couples were married and the remaining three were in informal relationships. Women and their partners were recruited at midwifery practices between September 2022 and July 2023. Participation in the study was voluntary; all respondents gave written informed consent to participate. The inclusion criteria were: age above 17 years and no current severe psychiatric disorders. Participants received remuneration in the form of money. 62% of women and 52% of men had higher education, 34% of women and 39% of men had secondary education, and 4% of women and 9% of men had elementary education. Participants lived in a village (38%), a medium-sized town (32%), or a big city (30%). 20% of the participants described their financial situation as very good, 60% as good, and 20% as average.

### Measures

The Fatalism scale of the Zimbardo Time Perspective Inventory (ZTPI-Fat; Zimbardo and Boyd, 2008) was used to measure the tendency to passively focus on the present connected with the belief that future is determined by fate. It consists of 10 items rated on a 5-point Likert-type scale. Cronbach’s alpha was .72 in this study.

The Dark Future Scale (DFS; Zaleski et al. 2019) was used to measure anxiety-based focus on the negatively evaluated future. This scale consists of 5 items rated on a 7-point scale. Cronbach’s alpha was .93 in this study.

The Edinburgh Postnatal Depression Scale (EPDS; Cox et al., 1987) was used to measure depressive symptoms. The scale consists of 10 items rated on a 5-point Likert-type scale. Cronbach’s alpha was .84 in this study.

The partner’s problematic SMU was measured using the Social Media Addiction Questionnaire (SMAQ; Hawi and Samaha 2017). The method consists of 8 items rated on a 7-point Likert-type scale. The items refer to different aspects of addiction, such as loss of control, or relapse and reinstatement. In this study, Cronbach’s alpha was .81.

### Procedure

The study was conducted in the first and the third trimester of pregnancy. Women were examined during their visits to the gynecologist’s office (a sociodemographic survey, pregnancy evaluation), and then they completed the online questionnaires (ZTPI, DFS, and EPDS). Men completed the online SMAQ. The study protocol was approved by the University Ethics Committee at the authors’ institution.

### Statistical analyses

The G*Power program (Erdfelder 2007) was used to determine the necessary sample size (t test, linear multiple regression fixed model, single regression coefficient, one tail, effect size ƒ² = .15, the alpha level set to .05, power set to .70) based on the average effect size in individual differences research (*r* ≈ .20; Gignac and Szodorai 2016). The results were analyzed using the SPSS 27 statistical package. Skewness and kurtosis were computed as indicators of the normality of variable distributions. To examine the associations of the partner’s problematic SMU with the woman’s time perspective and prenatal depressive symptoms, mediation analyses were performed. The results were found to be significant at *p* < .05.

## Results

The SMAQ, ZTPI, DFS, and EPDS scores were normally distributed. Descriptive statistics for the variables and the correlations between them in the first trimester are presented in Table 1.

**Table 1:** Correlations of studied variables in the first trimester

	1	2	3	4	5	6	7	8	9	10	11	12
1. women - age	-											
2. women – education	− .35*	-										
3. women - threatened miscarriage	− .28	.07	-									
4. men - age	.78***	− .25	-.29	-								
5. men – education	− .23	.37*	.37*	− .21	-							
6. couple’s marital status^	.10	− .05	− .26	.13	− .07	-						
7. couple’s financial situation	.04	− .12	− .61**	− .01	.40***	− .10	-					
8. couple’s place of living	− .12	.14	.15	.03	.14	− .11	.06	-				
9. women –EPDS	.01	.01	.05	− .11	.12	.07	.11	− .13	-			
10. women – ZTPI Fat	.09	.12	.17	− .02	.22	.03	.20	.04	.35**	-		
11. women – DFS	− .01	− .23	.28	− .12	.21	− .09	− .23	− .11	.33*	.39**	-	
12. men - SMAQ	− .13	− .04	.19	− .14	− .06	.02	− .14	− .11	− .16	− .22	.03	-
M	32.28	-	-	34.78	-	-	-	-	7.04.	23.17	13.74	16.80
SD	4.94	-	-	5.19	-	-	-	-	4.32	5.94	7.08.	8.74

Note. ^ 1 – unmarried, 2 – married; *p < .05; **p < .01; ***p < .001

The woman’s depressive symptoms in the first trimester were positively associated with fatalism and future negative perspective. Descriptive statistics and correlations for the third trimester are presented in Table 2. In the third trimester, women’s depressive symptoms correlated positively with their fatalism and future negative perspective and with their partners’ problematic SMU and negatively with their financial situation. The prospective analysis of correlations between variables measured in the first trimester and those measured in the third trimester showed no associations of the partner’s problematic SMU in the first trimester with the woman’s depressive symptoms, fatalism, and future negative perspective in the third trimester. To compare participants’ scores over time, we performed a paired samples *t*-test. The results revealed no differences in women’s depressive symptoms, fatalism, future negative perspective, and the partner’s problematic SMU between the first and third trimesters.

**Table 2:** Correlations of studied variables in the third trimester

	1	2	3	4	5	6	7	8	9	10	11	12
1. women - age	-											
2. women – education	− .35*	-										
3. women – threatened premature birth	− .12	.05	-									
4. men - age	.78***	− .25	− .12	-								
5. men – education	− .23	.37*	.05	− .21	-							
6. couple’s marital status^	.10	− .05	− .26	.13	− .07	-						
7. couple’s financial situation	.04	− .12	.04	− .01	.40***	− .10	-					
8. couple’s place of living	− .12	.14	.12	.03	.14	− .11	.06	-				
9. women –EPDS	− .07	.11	.20	− .15	− .09	− .15	− .37*	− .28	-			
10. women – ZTPI Fat	.03	.05	.06	− .01	.22	− .07	− .51**	.06	.49**	-		
11. women – DFS	− .12	− .02	.30	− .40	− .02	− .32	− .35	− .16	.77***	.58***	-	
12. men - SMAQ	− .20	− .19	.20	− .19	− .13	− .25	− .31	.14	.36*	.47**	.36*	-
M	32.28	-	-	34.78	-	-	-	-	7.00	22.79	14.24	16.04.
SD	4.94	-	-	5.19	-		-	-	4.81	6.62	6.62	8.32

Note. ^ 1 – unmarried, 2 – married; *p < .05; **p < .01; ***p < .001

**Fig. 1:**
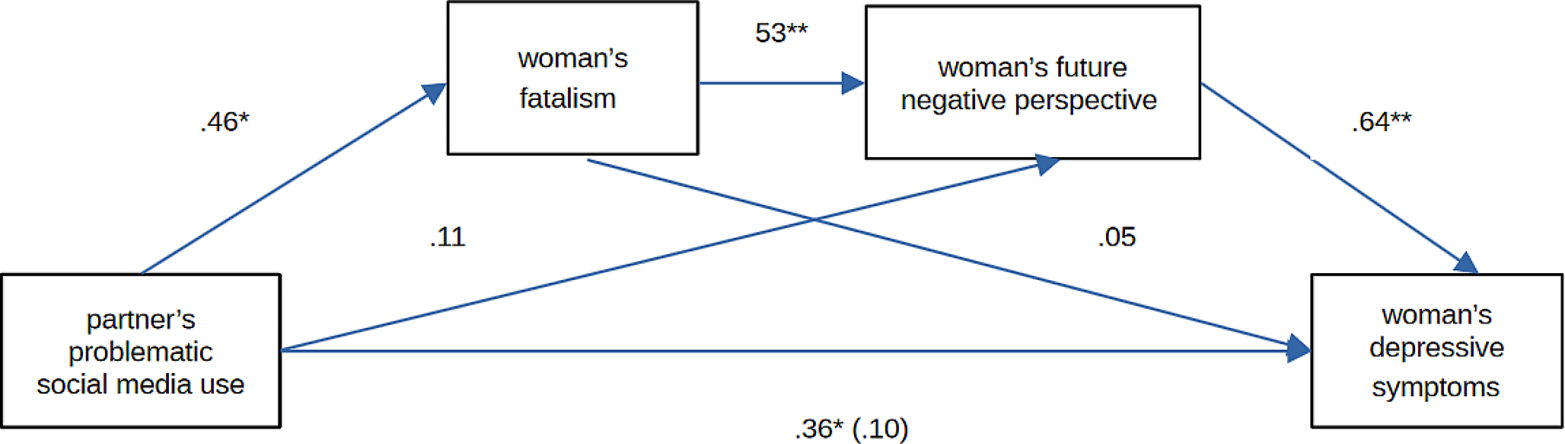
Serial multiple mediation model *p < .05; **p < .01

Mediation analyses were performed for the partner’s problematic SMU as a predictor, the woman’s depressive symptoms as a criterion, and the woman’s fatalism and future negative perspective as mediators (see Table 3). We performed analyses only for the third trimester variables, because only those variables significantly correlated with one another. Because the financial situation correlated significantly with the symptoms of depression in the third trimester, it was included in the analyses as a controlled variable (covariate). The results showed that in the third trimester the future negative perspective was a mediator of the relationship between the partner’s problematic SMU and the woman’s depressive symptoms. We conducted an additional mediation analysis with the partner’s problematic SMU as a predictor, the woman’s future negative perspective as a criterion, and the woman’s fatalism as a mediator, due to significant correlations between these variables. The results showed that the more the partner engaged in problematic SMU, the stronger the woman’s fatalism and, consequently, future negative perspective were. Based on these results, we entered the analyzed variables in a double mediation model (PROCESS Model 6; Hayes 2017; Fig. 1). The results showed that the more the partner engaged in problematic SMU, the stronger the woman’s fatalism was; this intensified the woman’s future negative perspective, which in turn led to an increase in the severity of her prenatal depressive symptoms in the third trimester of pregnancy (indirect effect: β = .16, *SE* = .09, 95% CI [.021, .393]).

**Table 3:** Results of mediation analyses for the partner’s problematic SMU as the independent variable in the third trimester

Mediator	Criterion	Direct			Indirect			Total		
		B	SE	95% CI	B	SE	95% CI	B	SE	95% CI
woman’s fatalism	woman’s depressive symptoms	.09	.11	− .135-.323	.10	.08	− .011-.299	.19	.10	− .020-.406
woman’s future negative perspective	woman’s depressive symptoms	.06	.08	− .111-.238	.13	.07	.015-.288	.19	.10	− .020-.406
woman’s fatalism	woman’s future negative perspective	.08	.14	− .204-.370	.18	.10	.027-.403	.26	.14	− .024-.550

## Discussion

This study aimed to examine the association of partner’s problematic SMU with the woman’s fatalism, future negative perspective, and prenatal depressive symptoms in the first and third trimesters of pregnancy. The participants were pregnant women and their male partners. The results indicate that the more intensively the partner engages in problematic SMU, the stronger the pregnant woman’s fatalism and, consequently, her future negative perspective are, which in turn leads to more severe prenatal depressive symptoms in the third trimester. This pattern can be interpreted as showing that the partner’s problematic SMU induces a sense of loss of control over events, a sense of helplessness, and a feeling of powerlessness in the woman (Abbasi 2019). The woman does not know what activities her partner engages in on social networking sites,. Combined with the awareness that attractive photos of other women are available on social networks (Reizer and Hetsroni 2014) and that her partner can easily contact them (Valenzuela et al. 2014), this can lead the pregnant woman to develop a sense of threat and a fear of what may happen. As the results of our research suggest, this pattern is probably especially strong in the last stage of pregnancy, when a woman’s body undergoes visible changes and when additional health problems often appear. The fear of danger intensifies the symptoms of prenatal depression. These results are consistent with research indicating a relationship between future anxiety and depression (Zaleski et al. 2019).

In addition, the partner’s time-consuming preoccupation with SMU may make the woman feel emotionally neglected and alone in coping with the difficulties of advanced pregnancy. The feeling of loneliness and lack of support from the partner may give rise to feelings of helplessness, powerlessness, and lack of influence on what may happen in the future. Research shows that what is of fundamental importance for a woman preparing for the role of a mother is a sense of security and the certainty that she can rely on her partner to help her (Cheng et al. 2016; Ngai and Ngub 2015).

The results of our research have some practical applications. They stress the importance of education about the negative consequences of the partner’s problematic SMU for the woman’s emotional functioning. The partner’s intensive SMU should be a warning sign, indicating the possibility of the woman’s emotional problems during pregnancy.

Several limitations of our research should be noted. Firstly, this study was a preliminary one, which is why the size of the sample was small. Due to the small size of the group, it is worth verifying the obtained results in subsequent studies on a larger group of couples expecting a child. Secondly, it should be remembered that our analyses were cross-sectional, which means the direction of the relationships could be also the opposite—for example, the woman’s bad mood could be a driver of the partner’s problematic SMU. It would be worth verifying these results in longitudinal studies in the third trimester of pregnancy to be able to determine with greater certainty the direction of the relationship between the analyzed variables. Moreover, variables such as the partner’s emotional state and the woman’s SMU were not analyzed. It would be worth taking these variables into account in future research.

## Conclusions

We found that the higher intensity of the partner’s problematic SMU was associated with the stronger the pregnant woman’s fatalism and future negative perspective, and with higher prenatal depressive symptoms in the third trimester. The results of our research show the possible mechanism explaining the association of the partner’s problematic SMU with the pregnant woman’s depressive symptoms. The results highlight the role of the woman’s fatalism and future negative perspective in this mechanism. Doctors caring for pregnant women, therapists and nurses conducting antenatal classes and other specialists in contact with expecting couples should pay more attention to the problem of the partner’s problematic SMU.
